# Soluble IL-2Rα correlates with imbalances of Th1/Th2 and Tc1/Tc2 cells in patients with acute brucellosis

**DOI:** 10.1186/s40249-020-00699-y

**Published:** 2020-07-13

**Authors:** Hua-Li Sun, Cheng-Jie Ma, Xiu-Fang Du, Si-Yuan Yang, Xiao Lv, Hong Zhao, Ling-Hang Wang, Yun-Xia Tang, Xing-Wang Li, Rong-Meng Jiang

**Affiliations:** 1 Department of Infectious Diseases, The Affiliated Hospital of Qingdao University, Qingdao, Shandong China; 2 The Laboratory of Infectious Diseases Centre, Beijing Di Tan Hospital, Capital Medical University, Beijing, China; 3 Department of Infectious Diseases, the Third People Hospital, Linfen City, Shanxi Province China; 4 Department of Laboratory Medicine, the Third People Hospital, Linfen City, Shanxi Province China; 5 Center for Infectious Diseases, Beijing Di Tan Hospital, Capital Medical University, Beijing, China

**Keywords:** Brucellosis, sIL-2Rα, Tc1, Tc2, Th1, Th2

## Abstract

**Background:**

Previous studies showed that soluble IL-2Rα is an important marker of cellular immune activation and might be a marker of treatment efficacy for children with brucellosis. However, data regarding adult patients with brucellosis were unknown. The aim of study was to explore the potential role of serum sIL-2Rα evaluating treatment responses in adult patients with brucellosis, and T cell immune status was also examined.

**Methods:**

During January 2016–April 2017, 30 patients with acute brucellosis from the Third People’s Hospital of Linfen in Shanxi Province and Beijing Di Tan Hospital, and 28 healthy controls were included in this study. Peripheral blood samples were collected before and after six weeks of antibiotic treatment. Serum sIL-2Rα levels were measured by enzyme-linked immunosorbent assay, and the percentage of Th1, Th2, Tc1, Tc2, and Tregs was detected by flow cytometry after intracellular staining for cytokines (interferon-γ and interleukin-4) and Foxp3 in T lymphocytes from peripheral blood. The obtained data were analyzed with Wilcoxon ranked sum tests for paired values, Mann-Whitney U-tests for comparisons between patients and healthy controls, and Spearman rank tests for correlation analyses.

**Results:**

Serum sIL-2Rα levels were significantly higher in patients than in controls (*P* = 0.001). A significant decline was observed in patients after the cessation of treatment (*P* < 0.001) and return to normal (*P* > 0.05). Th1, Tc1, Th2, and Tc2 cell frequencies were higher in patients than in healthy subjects (*P* < 0.05), while the Th1/Th2 and Tc1/Tc2 ratios were significantly lower (*P* = 0.0305 and 0.0005, respectively) and returned to normal levels after treatment. In patients with acute brucellosis, serum sIL-2Rα levels were negatively correlated with the Th1/Th2 ratio (*r* = − 0.478, *P* = 0.028), Tc1/Tc2 ratio (*r* = − 0.677, *P* = 0.001), and Tc1 percentage (*r* = − 0.516, *P* = 0.017). Serum sIL-2Rα and Tc2 percentages were positively correlated (*r* = 0.442, *P* = 0.045).

**Conclusions:**

Based on the correlations with Th1/Th2 and Tc1/Tc2 ratios, serum sIL-2Rα levels may reflect the immune response status. sIL-2Rα may be a marker for therapeutic efficacy in acute brucellosis.

## Background

Brucellosis, a re-emerging zoonotic infection worldwide, is a serious public health problem in many developing countries, with more than half a million new human infections each year [1]. The lack of an effective therapy or safe vaccine and tendencies towards chronicity and persistence [2] emphasize the importance of research focused on the immune response. *Brucella* is an intracellular pathogen that survives in macrophages. Host protection against *Brucella* depends on cell-mediated immunity, mainly involving activated antigen-presenting cells (macrophages and dendritic cells), CD4+ T helper cells (Th), and CD8+ cytotoxic T cells (Tc). The activation of monocyte macrophages and T lymphocytes induces a large number of immune molecules, including pro-inflammatory cytokines, anti-inflammatory cytokines, and chemokines, to coordinate the immune response, thereby determining disease progression and outcomes [3–6]. Studies have shown that mononuclear macrophages are activated with the release of soluble immunosuppressive molecules, such as sCD163 and sCD14, after *Brucella* infection, and changes in these immune molecules are expected to be effective indicators of treatment effects [7–9].

The soluble form of IL-2Rα (sIL-2Rα) is generated exclusively by the proteolytic cleavage of membrane IL-2Rα on activated T lymphocytes. IL-2R is composed of three subunits: α, β, and γ, and the IL-2Rα subunit is essential for high-affinity binding. sIL-2Rα exerts immunoregulatory effects via the regulation of T cell responses [10–12]. An elevated concentration of sIL-2Rα has been observed in autoimmune and inflammatory diseases [13], cancers [14], and various infectious diseases, including tuberculous [15–17]. Serum levels of sIL-2Rα are useful markers of disease progression. In children with brucellosis, elevated sIL-2Rα is associated with poor outcomes [18]. However, little is known about serum sIL-2Rα levels in adults with brucellosis.

In this study, we measured serum sIL-2Rα concentrations in adult patients with acute brucellosis before and after six weeks of antibiotic treatment to determine its prognostic value. It is well established that the Th1/Th2 and Tc1/Tc2 ratios can reflect cellular responses. To provide a theoretical basis for the clinical application of sIL-2Rα, we also investigated alterations in Th1/Tc1 lymphocytes (i.e., IFN-γ-producing lymphocytes) and Th2/Tc2 lymphocytes (i.e., IL-4-producing lymphocytes).

## Materials and methods

### Characteristics of the study population

In total, 30 patients with primary acute brucellosis (disease course < 6 months) older than 18 years and 28 healthy volunteers from the same area, matched by sex and age, were included in this study. In the brucellosis group, the disease course of 12 patients (40%) was less than 4 weeks, and the disease course of 18 patients (60%) was between 4 and 8 weeks. All patients had been treated in the Department of Infectious Disease of the Third People’s Hospital of Lin Fen in Shanxi Province and Beijing Di Tan Hospital between January 2016 and April 2017. Exclusion criteria for the healthy control subjects included acute/chronic diseases, smoking, medication, pregnancy, and any abnormalities in renal and liver function tests. Written informed consent was obtained from each subject and approval was obtained from the Ethics Committee of Beijing Di tan Hospital, Capital Medical University.

Brucellosis was diagnosed based on blood cultures and/or serologic tests according to current criteria [19]. Patients were excluded if they were pregnant, had evidence of auto-immune diseases, had other acute/chronic renal, liver, cardiovascular diseases, or received antibiotic treatment during the three months prior to the study. To avoid a Th2 bias in the immunologic studies, patients meeting the clinical criteria for atopy were excluded. Patients treated with immunosuppressants, immunomodulators, and/or other drugs capable of modifying the immune response during the three months preceding the study were also excluded.

All patients received the same antibiotic treatment regimen (ceftriaxone at 2 g/day for two weeks, doxycycline at 100 mg twice daily for six weeks, and rifampin at 600–900 mg/day for six weeks).

The symptoms and signs of all patients improved substantially or disappeared after the 6-week antibiotic regimen, with good outcomes and no relapse at six months after the treatment ended.

### Sample collection

Peripheral venous blood samples were collected before treatment and six weeks after treatment. The samples were stored in vacutainers (red top) without anticoagulant and EDTA-containing tubes and were used for serum and peripheral blood mononuclear cell (PBMC) isolation. The serum samples were obtained by centrifugation within four hours of blood collection and aliquots were stored at − 80 °C until use. PBMCs were isolated from EDTA blood by Ficoll-Paque (Amersham Pharmacia Biotech, Uppsala, Sweden) density gradient centrifugation. Cells were cryopreserved in fetal bovine serum (GIBCO, Grand Island, NY, USA) supplemented with 10% dimethylsulfoxide (DMSO) and stored in liquid nitrogen.

### Cytokine immunoassays

Serum levels of sIL-2Rα were measured in duplicate using a specific ELISA kit according to the manufacturer’s instructions (R&D Systems, Inc., Minneapolis, MN, USA). Serum samples require a 4-fold dilution prior to the assay. The minimum detectable doses of human sIL-2Rα for the test kits are 10 pg/ml, and the assay range is 78.0–5000 pg/ml. This range can be broadened by the dilution of samples with high concentrations. All assays were performed and read at an Infectious Diseases Centre using Gen5 3.0 (BioTek Synergy H1, Winooski, VT, USA). A four-parameter logistic curve was used to interpolate sample values based on the standard curve at a wavelength of 450 nm.

### Cell surface and intracellular cytokine staining

Cryopreserved PBMCs were used to evaluate cell viability by cell surface and intracellular cytokine staining using samples from 21 patients and 28 healthy controls. Cryopreserved PBMCs were thawed in RPMI 1640 medium (Invitrogen, Carlsbad, CA, USA), washed with phosphate-buffered saline (PBS) containing 1% bovine serum albumin (BSA), and incubated at room temperature for 20 min with the cell viability marker Fixable Viability Stain 510 (BD Biosciences, San Jose, CA, USA).

A lymphocyte phenotypic analysis was performed after staining with anti-CD4-PerCP-cy5.5 (BD Biosciences) and anti-CD8-APC-H7 antibodies. Intracellular IFN-γ and IL-4 staining was performed after stimulation with a leukocyte activation cocktail with BD Golgi Plug (BD Biosciences) for 5 h. The leukocyte activation cocktail contained PMA, ionomycin, and brefeldin A. Cells were initially stained with anti-CD4-PerCP-cy5.5 and anti-CD8-APC-H7, fixed, and permeabilized with Cytofix/Cytoperm according to the manufacturer’s instructions (BD Biosciences), followed by staining with anti-IFN-γ-FITC (BD Biosciences) and anti-IL-4-PE. To identify Tregs, freeze-thawed PBMCs were stained for surface molecules with anti-CD4-PerCP-cy5.5 and anti-CD25-PE-Cy7 antibodies and for intracellular molecules with an anti-FoxP3-AF647 antibody, in accordance with the manufacturer’s instructions (BD Biosciences).

All expression analyses were performed by flow cytometry using BD FACS Canto II with Diva (BD Biosciences). Forward scatter and side scatter light gating were used to exclude cell debris. Forward height and forward areas were used to exclude doublet cells. The final analysis was performed using FlowJo 10.0.7 (Tree Star Inc., Ashland, OR, USA).

### Statistical analyses

Statistical analyses and graphical presentation of results were performed using Graph Pad Prism 7.0 (San Diego, CA, USA). Data are expressed as median values and analyzed using Wilcoxon ranked sum tests for paired values (i.e., pre- and post-treatment for the same subject) or Mann-Whitney U-tests for between subject comparisons (i.e., patients versus healthy controls). Correlations were evaluated using the Spearman test. All tests were two-tailed, and *P*-values of less than 0.05 were considered statistically significant.

## Results

### Demographic, clinical, laboratory, and treatment data

A total of 30 patients (24 males [80%]) with acute brucellosis were enrolled, with an average age of 48 years (range: 20–69 years). We also enrolled 28 healthy controls (24 males [85.7%]), with an average age of 46 years (range: 24–69 years). There was no significant difference between patients and healthy controls with respect to gender or age (*P* > 0.05). With regards to the diagnosis of brucellosis, 16 patients (53.3%) had positive blood culture, the positive detection rate for the Rose-Bengal test was 100%, and the serum agglutination titers were more than 1/160 for all included patients. Common clinical symptoms were fever (83.3%), sweating (83.3%), weakness (76.7%), arthralgia and myalgia (53.3%), and anorexia (33.3%). Traditional inflammatory markers including C-reactive protein (CRP) and erythrocyte sedimentation rates (ESR) were also assayed by standard laboratory methods. In patients with acute brucellosis, CRP and ESR levels were significantly increased (Table S1). After the termination of standard treatment, symptoms and signs gradually disappeared, and CRP and ESR levels returned to normal and did not reoccur after six months of follow-up.

### Increased serum sIL-2Rα levels in patients with brucellosis decline upon treatment

**Fig. 1:**
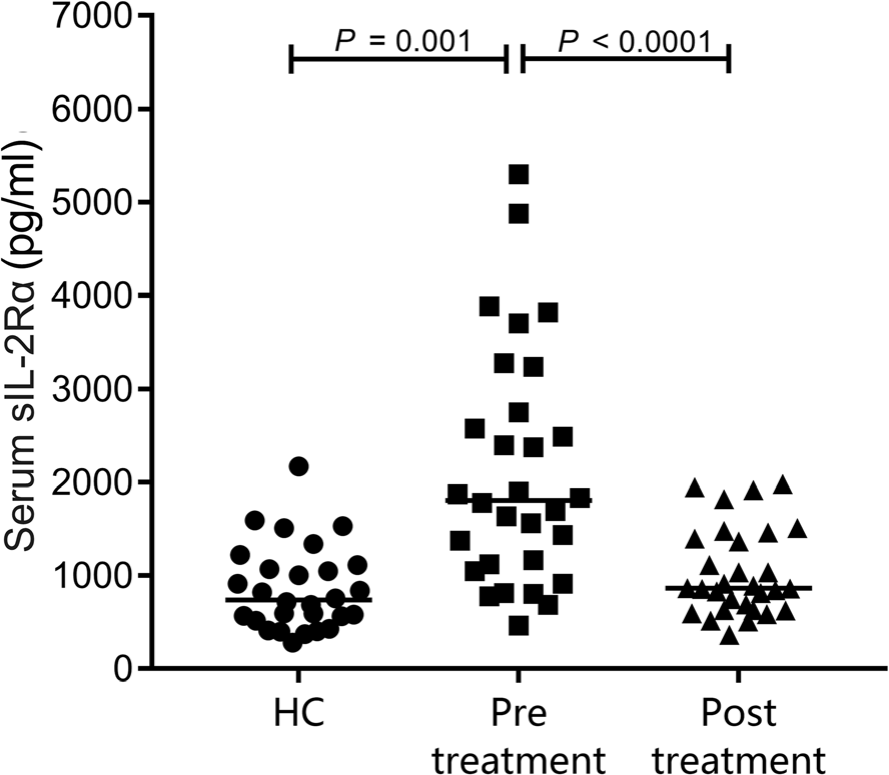
Serum sIL-2Rα levels for patients with acute brucellosis (pre- and post-treatment) and healthy controls. Circulating levels of sIL-2Rα in serum samples of patients with brucellosis (pre- and post-treatment, *n* = 30) compared to levels in healthy controls (*n* = 28), represented by dots. The horizontal line represents the median. A Mann-Whitney U-test was performed to evaluate the difference between controls and patients. Pre- and post-treatment levels were compared by the Wilcoxon matched-pair signed-rank test. sIL-2Rα: soluble Interleukin-2 receptor α; HC: healthy control

Serum sIL-2Rα levels in patients with acute brucellosis and in healthy controls are summarized in Fig. 1. The median (Q1–Q4) level of sIL-2Rα was 1807.14 (1104.66–2870.97) pg/ml in the pre-treatment group, 864.07 (630.82–1414.97) pg/ml in the post-treatment group, and 739.15 (528.41–1104.59) pg/ml in healthy controls. Median sIL-2Rα levels were significantly higher in patients with acute brucellosis than in healthy controls (Fig. 1, *P* = 0.001). After six weeks of treatment, serum sIL-2Rα levels decreased significantly (Fig. 1, *P* < 0.0001) and recovered to the levels in healthy controls (Fig. 1, *P* > 0.05).

### Increased percentages of Th1, Th2, Tc1, and Tc2 cells, and decreased Th1/Th2 and Tc1/Tc2 ratio in patients with brucellosis

**Fig. 2:**
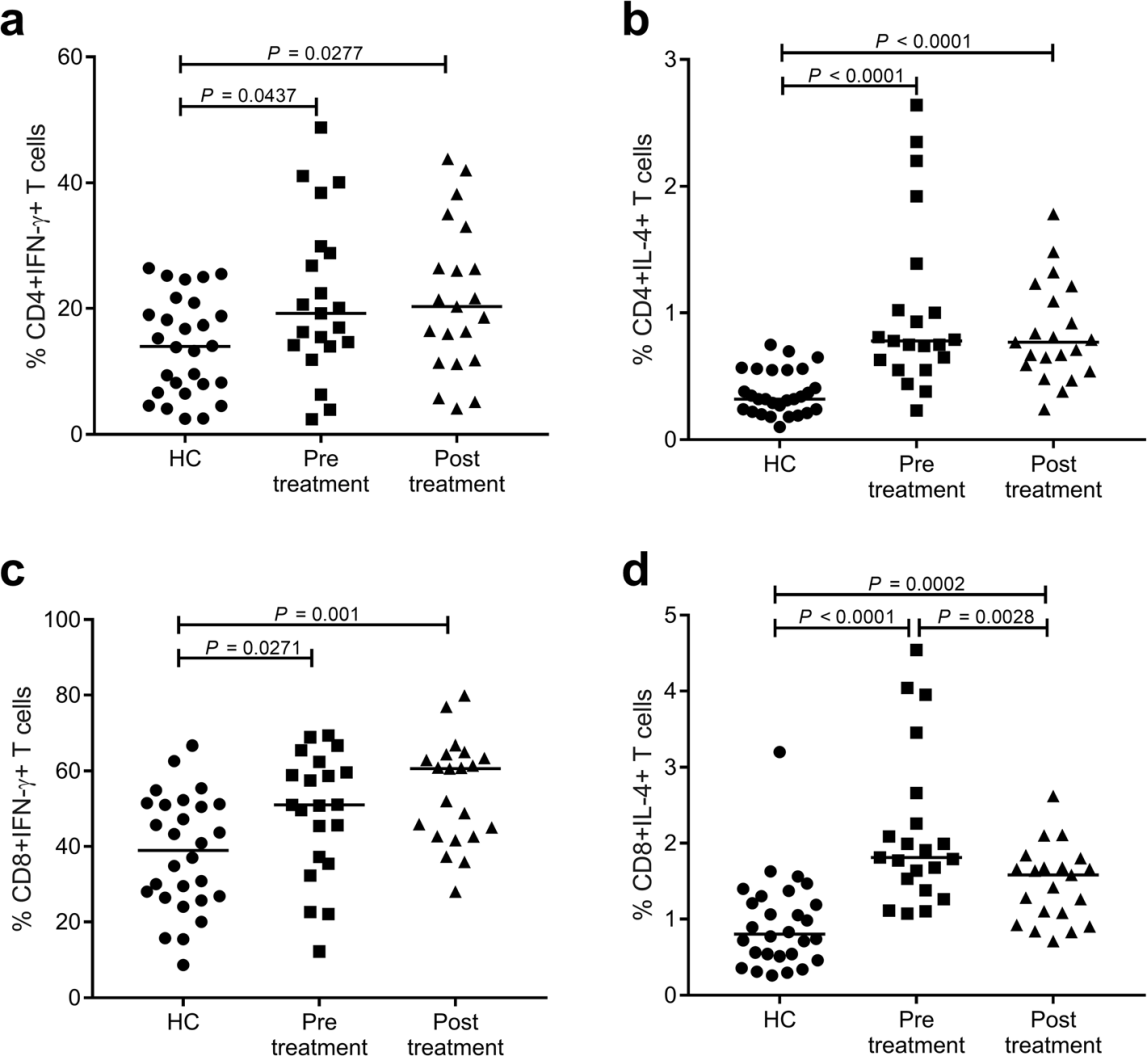
Percentages of Th1, Th2, Tc1, and Tc2 in samples from patients with acute brucellosis pre-and post-treatment and healthy controls. **a**, **b**, **c** and **d** Comparisons of the percentages of Th1, Th2, Tc1, and Tc2 cells (Th1: CD4 + IFN-γ + T cells, Th2: CD4 + IL-4 + T cells, Tc1: CD8 + IFN-γ + T cells, Tc2: CD8 + IL-4 + T cells) in healthy controls (HC) and patients with acute disease before treatment (Pre-treatment) and after treatment (Post-treatment) (*n* = 28, 21, and 21, respectively) are shown. The vertical line represents the median. A Mann–Whitney U-test was performed to compare controls and patients, and pre- and post-treatment values for patients were compared by the Wilcoxon matched-pair signed-rank test. HC: healthy control

To investigate changes in CD4+ and CD8+ T cell subsets in patients with acute brucellosis, we analyzed the following subsets in the blood: CD4 + IFN-γ + (Th1), CD4 + IL-4+ (Th2), CD8 + IFN-γ + (Tc1), and CD8 + IL-4+ (Tc2) (Fig. 2 and Table 1). Lymphocytes were gated based on a forward vs side scatter dot plot, and then, we analyzed different CD4+ and CD8+ T cell subsets based on gated cells. A representative example of the gating strategy is illustrated in Figure S1. Peripheral blood lymphocytes from 21 patients with brucellosis and 28 healthy controls were evaluated. As shown in Fig. 2a, b and Table 1, percentages of Th1 (14.1–29.4%) and Th2 (0.6–1.2%) were both markedly higher in patients than in healthy controls (7.0–20.4% and 0.2–0.6%, respectively; *P* = 0.0437 and *P* < 0.0001, respectively). Similar to Th1 and Th2 cells, the Tc1 (36.3–61.0%) and Tc2 percentages (1.5–2.5%) were both significantly higher in patients than in controls (26.5–51.2% and 0.5–1.3%, respectively) (Table 1 and Fig. 2c, d; *P* = 0.0271 and *P* < 0.0001, respectively). However, further analyses indicated that the Th1/Th2 and Tc1/Tc2 ratios were significantly lower in patients than in healthy controls (Table 1; *P* = 0.0305 and *P* = 0.0005, respectively), and there was a significant bias toward both Th2 and Tc2 cell responses in patients with acute brucellosis. At the end of the treatment period, both Th1/Th2 and Tc1/Tc2 ratios returned to normal levels (Table 1; *P* > 0.05). Furthermore, as Treg cells with negatively regulatory effects on immunity, CD4 + CD25 + FoxP3 + T cells were evaluated. As shown in Table 1, although the proportion of Tregs was higher in patients, the difference between groups was not significant.

**Table 1:** Percentages of Th1, Th2, Tc1, and Tc2 from acute brucellosis samples pre-and post-treatment and healthy controls

	Pre-treatment (*n* = 21) Median (Q1–Q3)^f^	Post-treatment (*n* = 21) Median (Q1–Q3)^f^	Healthy control (*n* = 28) Median (Q1–Q3)^f^	*P*-value^a^ pre- vs post-treatment
CD4 + IFN-γ+	19.2 (14.1–29.4) *	20.3 (11.6–29.7) *	14.0 (7.0–20.4)	ns^b^
CD4 + IL-4+	0.8 (0.6–1.2) *	0.8 (0.6–1.2) *	0.3 (0.2–0.6)	ns
CD8 + IFN-γ+	51.0 (36.3–61.0) *	60.6 (42.7–63.9) *	39.0 (26.5–51.2)	ns
CD8 + IL-4+	1.8 (1.5–2.5) *	1.6 (1.0–1.7) *	0.8 (0.5–1.3)	0.0028
Treg^c^	1.4 (0.9–2.1)	2.0 (1.2–2.5)	1.2 (0.8–2.2)	0.093
Th1/Th2 ratio^d^	22.6 (15.8–28.7)*	23.8 (18.4–32.7)	37.4 (19.2–71.4)	ns
Tc1/Tc2 ratio^e^	30.4 (14.1–35.0) *	39.0 (30.5–45.4)	50.7 (31.0–73.7)	0.0006

^a^Data was analyzed using Wilcoxon matched-pair signed-rank test

^b^ns, no significant difference

^c^Treg, proportion of CD4^+^CD25^+^FoxP3^+^ T cells among CD4^+^ cells

^d^Th1/Th2 ratio, ratio of peripheral CD4 + T cells producing IFN-γ (CD4 + IFN-γ+) to those producing IL-4 (CD4 + IL-4+)

^e^Tc1/Tc2 ratio, ratio of peripheral CD8 + T cells producing IFN-γ (CD8 + IFN-γ+) to those producing IL-4 (CD8 + IL-4+)

^f^Q1 and Q3 represent the first and third quartiles, respectively

*Significant difference (*p* < 0.05) in comparison with healthy controls

### Correlation between serum sIL-2Rα levels and the percentage of Th1, Th2, Tc1, and Tc2 cells in patients with brucellosis

**Fig. 3:**
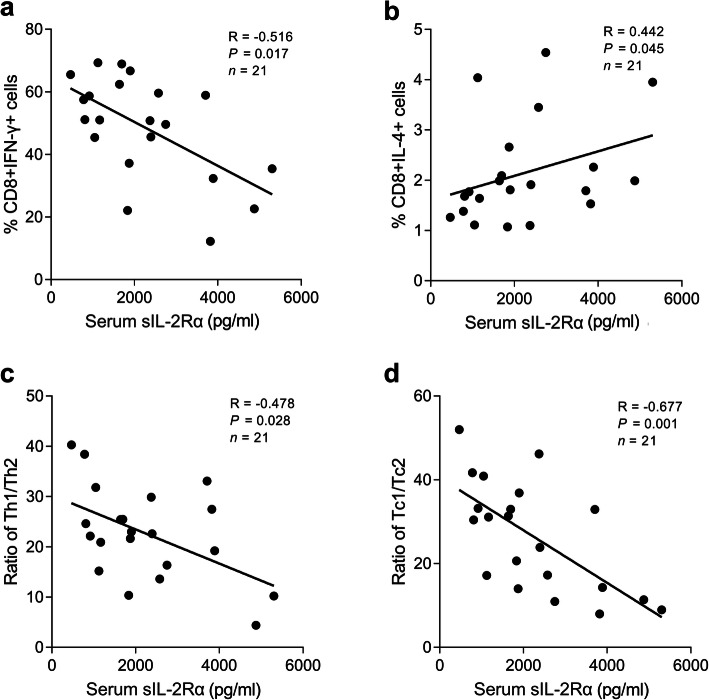
Correlations between serum sIL-2Rα levels and the percentage of Tc cells, Th1/Th2 ratio, and Tc1/Tc2 ratio in patients with brucellosis (*n* = 21)

For a more detailed analysis, we explored the correlations between serum sIL-2Rα levels and cell subsets (Table 2 and Fig. 3). Serum sIL-2Rα levels were inversely associated with the percentage of Tc1 cells (Fig. 3a; *r* = − 0.516, *P* = 0.017), Th1/Th2 ratio (Fig. 3c; *r* = − 0.478, *P* = 0.028), and Tc1/Tc2 ratio (Fig. 3d; *r* = − 0.677, *P* = 0.001) in patients with acute brucellosis. In contrast, serum sIL-2Rα levels were positively correlated with the percentage of Tc2 cells (Fig. 3b; *r* = 0.442, *P* = 0.045). At the end of treatment period, the relationships disappeared, with normalized sIL-2Rα levels. Surprisingly, no correlation was found between serum sIL-2 levels and proportions of Tregs.

**Table 2:** Correlations between serum sIL-2Rα levels and percentages of Th1, Th2, Tc1 and Tc2 in acute adult brucellosis (*n* = 21)

	*R*	*P*-value
CD4 + IFN-γ+	−0.343	0.128
CD4 + IL-4+	0.001	0.996
CD8 + IFN-γ+	−0.517	0.017
CD8 + IL-4+	0.442	0.045
CD4 + CD25 + FoxP3 + T cell	−0.208	0.366
Th1/Th2 ratio	−0.478	0.028
Tc1/Tc2 ratio	−0.677	0.001

*R*, Spearman correlation coefficient

Th1, CD4 + IFN-γ+; Th2, CD4 + IL-4+; Tc1, CD8 + IFN-γ+; Tc2, CD8 + IL-4+

## Discussion

We investigated serum levels of the immune-inhibitory molecule sIL-2Rα, frequencies of Th1, Th2, and cytotoxic (Tc1 and Tc2) T-lymphocyte subsets in PMA and ionomycin-stimulated PBMCs as well as the frequency of Tregs in adult patients with brucellosis before and after the standard 6-week course of antibiotics. Our data agree with previous results showing that sIL-2Rα levels are increased in patients with brucellosis. Furthermore, serum sIL-2Rα levels were inversely related to the Th1/Th2 ratio (especially the Tc1/Tc2 ratio) during the acute phase of adult brucellosis, suggesting that serum sIL-2Rα could serve as an indicator of the immune state in *Brucella* infection. After effective treatment, serum sIL-2Rα levels and Th1/Th2 and Tc1/Tc2 ratios returned to normal, indicating an improvement in host resistance. These findings further suggest that serum sIL-2Rα is a potentially effective marker of treatment responses in adult patients with brucellosis.

In a murine model, protective acquired immunity to *Brucella* involves the T cell-dependent activation of macrophages involving both CD4+ and CD8 + T cells. It is well established that the clearance of *Brucella* requires IFN-γ-mediated type-1 T cell (Th1 and Tc1) immune responses [20–22], and the IL-4, IL-10, Th2 response determines susceptibility to *Brucella* and disease severity [23–25]. As another T-cell subset, Tregs have a pivotal role in the regulation of the immune response to *Brucella*. An in vivo experiment demonstrated that the production of IL-10 by CD4 + CD25 + T cells is critical for the modulation of macrophage function during early *Brucella* infections [26]. In a BALB/c mouse model of brucellosis, CD4 + CD25 + T regulatory cells may limit the effectiveness of CD4 + T cells and favor the maintenance and progression of *B. abortus* infection [27].

IL-2Rα is expressed on many hematopoietic cells, including subsets of T and B cells (e.g., regulatory T cells), and is upregulated by the activation of these cells. Upon activation, immune cells proliferate and release sIL-2Rα in vitro [28, 29]. Therefore, measurable serum sIL-2Rα levels are thought to reflect sustained immune activation during infection or inflammation and have been used as a biomarker to characterize disease progression, prognosis, and treatment. In a previous study of 20 children with brucellosis [18], increases sIL-2Rα levels were reduced after the 6-week antibiotic regimen in 17 children who subsequently had good outcomes without relapse. In three patients with relapse, serum sIL-2Rα levels did not decline significantly after the termination of treatment. Our sIL-2Rα assay results for adults confirmed previous observations that sIL-2Rα is significantly elevated in patients with brucellosis. However, our data showed that serum sIL-2Rα levels return to normal at the end of the treatment period. The differences between studies may be explained by differences in medications; in our study, the regimen was doxycycline and rifampin for six weeks plus ceftriaxone for two weeks, while the previous study used a combination of oral rifampin and trimethoprim-sulfamethoxazole for six weeks. Unfortunately, due to the small sample size, we did not capture relapses, which would weaken the importance of sIL-2Rα as a prognostic marker in adult patients with brucellosis.

An imbalance between the production of Th1 and Th2 (Tc1 and Tc2) cytokines contributes to an impairment in T cell-mediated immunity, which may ultimately influence anti-bacterial immune responses. In our study, we observed imbalances between Th1 and Th2 as well as between Tc1 and Tc2, which have been reported with various intracellular bacterial infections, including tuberculosis [30, 31]. Our findings revealed higher percentages of Th1, Tc1, Th2, and Tc2 lymphocytes in patients with brucellosis as compared than in healthy controls, which consistent with previous results [32]. Type 1 and type 2 immune responses are induced simultaneously in the acute phase. Our results also indicated that the Th1/Th2 and Tc1/Tc2 ratios are significantly decreased in the acute phase, reflecting a general skew in the immune response away from type-1 and toward type-2, which is associated with the suppression of the immune response; this supports the concept that a predominantly type-2 cell response is associated with the pathogenesis of brucellosis. However, Rafiei et al. found that the Th1 response is dominant in early brucellosis, with a shift toward the Th2 phenotype as the disease progresses [33]. This difference among studies might be due to the difference in patient recruitment, since the previous study included patients who had already received antibiotic treatment. In the early stage of infection in mouse models, *B. abortus* induces the expression of low levels of proinflammatory cytokines and high levels of anti-inflammatory cytokines [26, 34, 35]. Our findings, in conjunction with previous investigations, suggest that immunological changes in Th1/Th2 or Tc1/Tc2 ratios may promote an immunosuppressive condition in patients with acute brucellosis and a limited inflammatory response develops in the early phase.

To further clarify the immunological basis of sIL-2Rα as a prognostic marker, we analyzed the correlation between serum sIL-2Rα levels and T cell immune parameters in patients with acute brucellosis. We found the first evidence for negative correlations between serum sIL-2Rα levels and Th1/Th2 and Tc1/Tc2 ratios, supporting the immunosuppressive effect of sIL-2Rα during *Brucella* infection. Interestingly, serum sIL-2Rα levels were only associated with Tc1 and Tc2 cell frequencies, but not CD4 + T cells. At the end of the treatment period, the serum sIL-2Rα levels, Th1/Th2 ratio, and Tc1/Tc2 ratio return to normal. These results establish several important points. First, serum sIL-2Rα levels might reflect peripheral blood T-cell profiles and could serve as an indicator of the immune state, which is closely related to prognosis. Second, the strong correlation between serum sIL-2Rα levels and CD8 + T cell subsets, but not CD4 + T cells, implied that CD8 + T cells play a more prominent role in the control of *Brucella* infection. Recent studies have shown that defects in *Brucella*-responsive CD8(+) T cells allow the persistence of infection [21, 22, 36]. Therefore, we speculated that serum sIL-2Rα levels contribute to the down-regulation of CD8 + T cells in patients with brucellosis. Although a detailed functional analysis of the role in sIL-2Rα in regulating the immune response in vitro is still needed, several recent studies have made progress in our understanding of the sIL-2Rα-mediated suppressive mechanism. In lymphoma, sIL-2Rα may present IL-2 in vitro to CD4 + T cells and cause the differentiation of these cells into FoxP3 + Treg cells, resulting in the suppression of CD8 + T cells [11]. We also study changes in Treg (CD4 + CD25 + FoxP3 + T) cells and did not detect a correlation between Treg cells and sIL-2Rα. Hence, the specific immunological mechanism of serum sIL-2Rα requires further study. An unexpected discovery was that although the percentage of Treg cells was elevated in patients, there was no significant difference between patients and healthy controls, different from the results of a previous study showing that Treg cells are increased in patients and decline with therapy [37]. The sample size, patient characteristics, and antibiotic may explain the difference.

Despite the meaningful observations, our study has some limitations, including the small sample size and few time points measured. The prognosis of brucellosis was correlated with clinical staging; research with a larger sample size, more follow-up time points, and analyses from different clinical stages will help to better explain sIL-2Rα as a beneficial laboratory test. Additionally, the present study only showed an association between sIL-2Rα and CD8 + T cell subpopulations in acute patients with brucellosis, and mechanistic research involving sIL-2Rα-mediated immune suppression is needed to better characterize the relationship between sIL-2Rα and CD8 + T cell immunity.

## Conclusions

These data demonstrate that serum sIL-2Rα levels increase during acute infection but return to normal levels during recovery. There is a strong negative correlation between increased serum sIL-2Rα levels and the Tc1/Tc2 ratio during the acute phase. Based on these results, serum sIL-2Rα levels could serve as an indicator of the immune suppressive state in *Brucella* infection and is a candidate marker of treatment responses in adult patients with brucellosis. Further investigations with a larger sample sizes and longer follow-up to capture relapses will strengthen the clinical value of sIL-2Rα.

## Supplementary information

**Additional file 1: Figure S1.** Representative gating strategy for different CD4+ and CD8 + T cell subsets. In this sample gating, cells were first gated for lymphocytes (SSC-A vs FSC-A). The lymphocytes were gated based on single cells and live cells and were further analyzed for IFN-γ-positive, IL-4 CD4 + T, and CD8 + T cells.

**Additional file 2: Table S1.** Serum sIL-2Rα and CRP, ESR levels of patients with acute brucellosis in pre-and post-treatment and healthy controls.

## Data Availability

Not applicable.

## Abbreviations

ELISA: Enzyme-linked immunosorbent assay
IFN-γ: Interferon-γ
IL-4: Interleukin-4
Th: T helper cells
Tc: CD8+ cytotoxic T cells
sIL-2Rα: Soluble Interleukin-2 receptor α
PBMC: Peripheral Blood mononuclear cell
PBS: Phosphate-buffered saline
BSA: Bovine serum albumin
CRP: C-reactive protein
ESR: Erythrocyte sedimentation rate
DMSO: Dimethylsulfoxide
