# Supplementary figures and images for: Soluble IL-2Rα correlates with imbalances of Th1/Th2 and Tc1/Tc2 cells in patients with acute brucellosis
Source: Infect Dis Poverty. 2020 Jul 13;9:92. doi: 10.1186/s40249-020-00699-y (PMC7359011; doi:10.1186/s40249-020-00699-y)

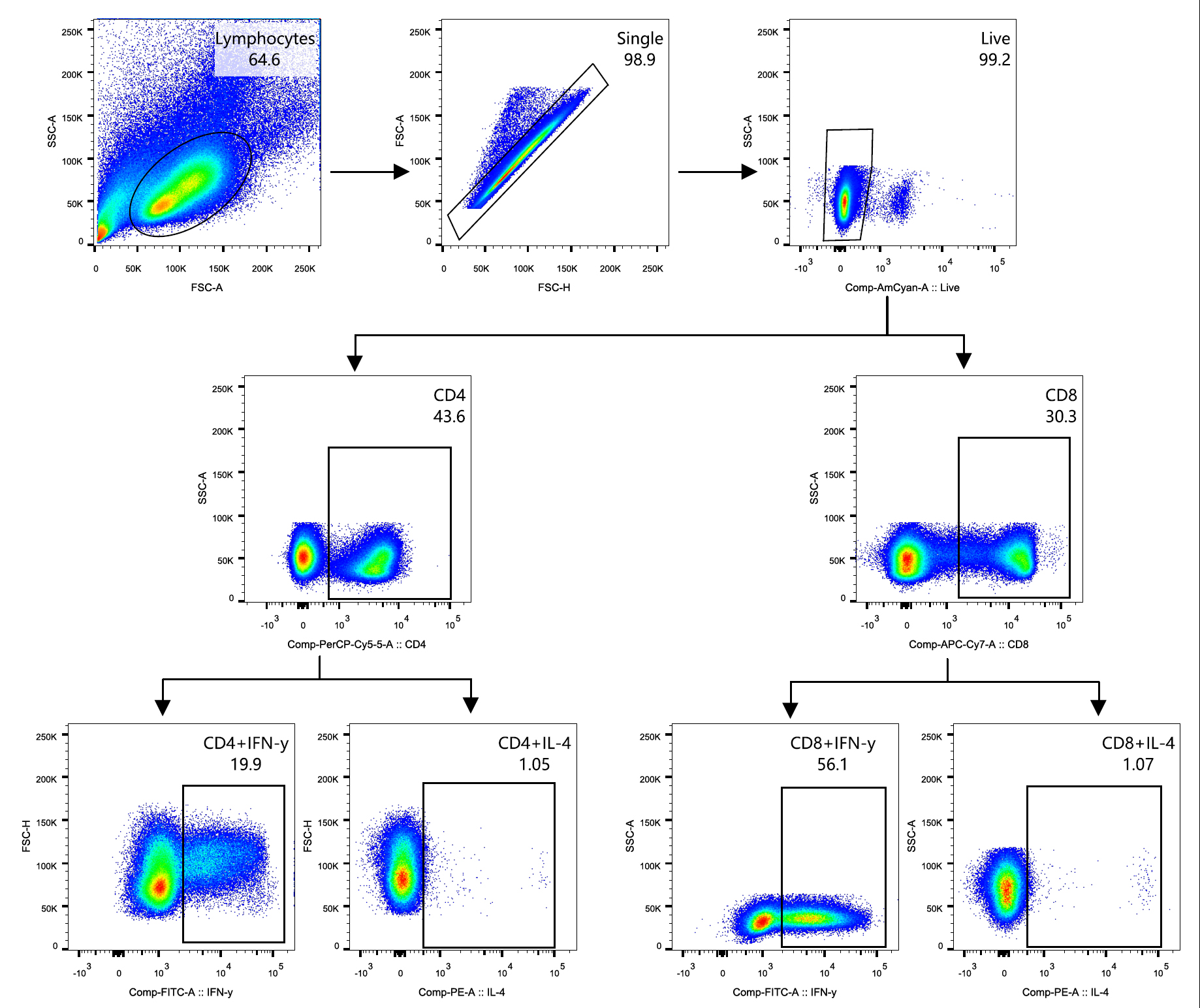

Supplement: Supplementary file 1 — Additional file 1: Figure S1. Representative gating strategy for different CD4+ and CD8 + T cell subsets. In this sample gating, cells were first gated for lymphocytes (SSC-A vs FSC-A). The lymphocytes were gated based on single cells and live cells and were further analyzed for IFN-γ-positive, IL-4 CD4 + T, and CD8 + T cells. [file 40249_2020_699_MOESM1_ESM.jpg]
