# Supplementary material for: Soluble IL-2Rα correlates with imbalances of Th1/Th2 and Tc1/Tc2 cells in patients with acute brucellosis
Source: Infect Dis Poverty. 2020 Jul 13;9:92. doi: 10.1186/s40249-020-00699-y (PMC7359011; doi:10.1186/s40249-020-00699-y)
Supplement: Supplementary file 2 — Additional file 2: Table S1. Serum sIL-2Rα and CRP, ESR levels of patients with acute brucellosis in pre-and post-treatment and healthy controls. [file 40249_2020_699_MOESM2_ESM.docx]

**Table S1. Serum sIL-2Rα and CRP, ESR levels of patients with acute brucellosis in pre-and post-treatment and healthy controls.**

|  | Pre-treatment  (n = 30)  Median (Q1–Q3)^b^ | Post-treatment  (n = 30)  Median (Q1–Q3)^b^ | Healthy controls  (n = 28)  Median (Q1–Q3)^b^ | P-value^a^ pre vs. post treatment |
| --- | --- | --- | --- | --- |
| sIL-2Rα(pg/mL) | 1807.14 (1104.66–2870.97)* | 864.07 (630.82–1414.97) | 739.15 (528.41–1104.59) | ＜0.0001 |
| CRP(mg/L) | 18.78（7.23-30.99）* | 3.08（2.02-3.95） | 1.80（0.79-3.73）^c^ | ＜0.0001 |
| ESR(mm/h) | 36.5（17.5-62.75）* | 10.0（7.0-12.25） | 6.5（3.0-11.0） | ＜0.0001 |

^a^Data was analyzed using Wilcoxon matched-pair signed-rank test.

^b^Q1 and Q3 represent the first and third quartiles, respectively.

^C^Data of three sample is invalid (CRP＜0.5mg/L), twenty-five samples were collected.

*Significanttdifference (p < 0.05) in comparison with healthy controls.

CRP: C-reactive protein; ESR: erythrocyte sedimentation rate.
